# Diagnostic and Therapeutic Potential of microRNAs in Acute Kidney Injury

**DOI:** 10.3389/fphar.2020.00657

**Published:** 2020-05-14

**Authors:** Timo Brandenburger, Johan M. Lorenzen

**Affiliations:** ^1^Department of Anesthesiology, University Hospital Duesseldorf, Duesseldorf, Germany; ^2^Division of Nephrology, University Hospital Zurich, Zurich, Switzerland

**Keywords:** acute kidney injury, microRNA, noncodingRNA, kidney, theranostics

## Abstract

During hospital stay, about 20% of adult patients experience an episode of acute kidney injury (AKI), which is characterized by a rapid decrease in kidney function. Diagnostic tools regarding early diagnosis of kidney dysfunction prior to AKI and markers of renal recovery are not available. Additionally, there is no therapeutic option for the treatment of AKI. Thus, better and more specific diagnostic and therapeutic options are urgently needed in daily clinical practice. NoncodingRNAs (ncRNAs) have come into focus of research in the context of AKI in the last decade. The best characterized group of ncRNAs are microRNAs (miRNAs). An increasing body of literature has shown that miRNAs are involved in the pathogenesis of AKI and that they are promising future tools in the diagnosis and therapy of AKI. However, there are obstacles to be overcome before miRNAs can be transferred to patient care. This review will give an overview of our current knowledge of miRNA involvement in the context of AKI while critically evaluating their diagnostic and therapeutic potential.

## Introduction

According to Kidney Disease: Improving Global Outcomes (KDIGO), acute kidney injury (AKI) is diagnosis involves elevated levels in creatinine and/or a decrease in urine output (KDIGO, 2012). AKI occurs in up to 50% of critically ill patients (Hoste et al., 2015; Chawla et al., 2017). Its etiology is diverse and involves patient susceptibility (e.g., age, known CKD, diabetes mellitus) as well as exposure to potentially harmful factors contributing to the development of AKI (e.g., sepsis, burns, nephrotoxic drugs, cardiac surgery). AKI remains a major health issue in critically ill patients (Hoste et al., 2015; Chawla et al., 2017). It is an important factor of short-term patient outcome as it can cause electrolyte disorders, fluid accumulation, and acid–base derangement. Indeed, mortality in critically ill patients that experience an episode of AKI raises up to 50% in those that require renal replacement therapy (RRT) (Ympa et al., 2005). AKI furthermore goes along with an increase of cardiovascular events (Chawla et al., 2014a) and the development of CKD (Venkatachalam et al., 2010; Chawla et al., 2014b). There are no specific treatment options in daily clinical practice besides renal replacement therapy (RRT). Morbidity and mortality of AKI patients remains high.

The high incidence of AKI as well as the impairment in outcome indicates that there is an urgent need for new markers and therapeutic options in the prevention and treatment of AKI.

To date, diagnostic markers of AKI are insufficient and late. Therapeutic options are limited to renal replacement therapy. Therefore, noncoding RNAs (nc RNAs) have come into focus of research in the last decade as potential markers and therapeutic targets in AKI. The best investigated class of ncRNAs in this context is microRNAs (miRNAs).

MiRNAs are small ncRNAs that regulate gene expression post transcriptionally (Ambros, 2001). They repress protein translation or induce degradation of messengerRNA (mRNA) (Bartel, 2009) through base-pairing with complementary sequences of the 3´untranslated region (UTR) of mRNAs. A high number of the more than 2000 identified miRNAs is highly conserved between species (www.mirbase.org). In the canonical pathway, miRNAs are transcribed as primary miRNAs (primiRNA) and then cleaved by the enzymes Drosha/Dgcr8 resulting in the so-called premiRNA (Friedman et al., 2009). The enzyme Dicer cleaves the premiRNA which finally results in the mature miRNA with a length of 20-24 nucleotides. The two strands of the mature miRNA are then separated and incorporated into the miRNA induced silencing complex (miRISC) (Bartel, 2009). Within the miRISC, miRNAs bind to the 3'-untranslated region of mRNAs thereby inhibiting translation of messenger RNAs or inducing degradation of mRNAs. In both cases, miRNAs action results in a reduction of protein translation (Baek et al., 2008). In the non-canonical pathway, miRNAs do not originate from their own genes. Instead, they are excised from other larger RNAs, such as tRNAs, introns or small nucleolar RNAs.

It has been shown that miRNAs are essential players in physiological processes from development to death of cells, tissues, and organisms. Moreover, miRNAs are involved in pathophysiological processes including diseases of the kidney. MiRNAs are stable in body fluids (such as plasma and urine) (Gilad et al., 2008) and can be detected easily. These characteristics make them an interesting class of molecules as novel diagnostic markers of myocardial infarction (Goretti et al., 2014), lung cancer (Vencken et al., 2015) or breast cancer (Chan et al., 2013). MiRNA function can be manipulated by the application miRNA-agonists (so-called mimics) or antagonists. They might, therefore, also be future therapeutic targets in the treatment of AKI.

A high number of miRNAs are expressed in mammalian kidneys. A study from 2004 showed five miRNAs that are enriched in human kidneys including miR-192, -194, -215, -216, and miR-204 (Sun et al., 2004). A newer study from 2016 showed miR-449c-5p, -449b-5p, and -449a to be expressed especially in kidneys (Ludwig et al., 2016). It is known that the expression of these miRNAs and others is altered in renal pathologies such as chronic kidney disease (CKD). Several studies have shown that the expression of miRNAs in CKD decreases in higher stages of the disease (Neal et al., 2011). These miRNAs might then be involved in pathological remodeling processes such as abnormal vascularization (Chen et al., 2013).

Besides the well documented involvement of miRNAs in CKD, it has been shown that miRNAs play an essential role in the pathology of AKI as well. Indeed, various miRNAs have been shown to be dysregulated in AKI in animal models as well as in human studies. A number of preclinical and clinical studies have focused on the role of miRNAs in the context of AKI. This review will give an overview of the current knowledge of miRNAs in the context of AKI as well as a perspective of miRNAs as diagnostic and/or therapeutic tools in AKI diagnosis and treatment.

### MiRNAs in Experimental AKI Studies

#### Experimental AKI Studies: Diagnostic Potential of miRNAs

To date, standard clinical markers of renal function are creatinine and urea. Additionally, a reduction in diuresis (oliguria, anuria) is one of the AKI signs. Especially creatinine, the most common marker of renal function, is an unspecific and late marker of AKI (Bagshaw et al., 2008). Oliguria, on the other hand, is influenced by multiple factors such as volume deficiency, heart failure or diuretics.

A large number of potential new AKI biomarkers have been extensively evaluated in the last years (reviewed in (Teo and Endre, 2017)). The most relevant markers of these are neutrophil gelatinase-associated lipocalin (NGAL), the cysteine protease inhibitor Cystatin C, kidney injury molecule 1 (KIM-1) and the product of insulin like growth factor binding protein 7 (IGFBP-7) and tissue inhibitor of metalloproteinase 2 (TIMP-2). With areas under the curve (AUC) of 0.67 for NGAL (Han et al., 2009), 0.71 for cystatin C (Koyner et al., 2008), 0.65 for KIM-1 (Han et al., 2009), and 0.80 for the product of IGFBP7 and TIMP-2 (Kashani et al., 2013), these markers perform better than the standard markers creatinine and urea. To date, most of these markers have not been established in clinical practice. A large number of miRNA-studies have, therefore, analyzed the potential of miRNAs as markers and/or potential therapeutics in AKI over the last decade including animal as well as human studies. The most relevant miRNAs as potential markers of AKI are summarized in **Table 1**.

**Table 1:** Selection of experimental miRNA studies focusing on biomarker potential in AKI.

MiRNA	Effect	AKI model	Origin of sample	Reference
**MiR-16**	Renal function is attenuated in AKI mice by overexpression of miR-16	Unilateral renal ischemia in mice	Kidney tissue and urine	Chen et al., 2016
**MiR-21**	It is upregulated in kidneys after warm ischemia in mice	Unilateral renal ischemia in mice	Kidney tissue	Godwin et al., 2010
	Urinary miR-21 is a marker of hypertensive kidney injury in rats	Hypertensive mice with renal tubular lesion	Urine	Chen et al., 2017
	MiR-21 and the miR-17-family are activated after I/R injury in mice	Lethal and sublethal ischemia in mice	Kidney tissue	Kaucsar et al., 2013
	Upregulated in rat kidneys after I/R injury	I/R injury or gentamicin in rats	Plasma and urine	Saikumar et al., 2012
**Mir-30a, and -e, miR-188**	Levels of these miRNAs are upregulated in plasma after contrast induced AKI in rats	Contrast induced AKI in rats	Plasma, kidney tissue	Sun et al., 2016
**MiR-34a**	In mice, cisplatin treatment induced the expression of miR-34a is induced in renal tissue after cisplatin induced AKI in mice	Cisplatin induced AKI in mice	Kidney tissue	Bhatt et al., 2010
**MiR-125b**	Significantly increased in rat kidneys after I/R injury	I/R injury in rats	Kidney tissue	Guclu et al., 2017
**MiR-140-5p**	Upregulated in kidneys. Targets the transcription factor NRF2.	Cisplatin induced AKI in mice	Kidney tissue	Liao et al., 2017a; Liao et al., 2017b
**MiR-146b**	Serum levels of miR-146b increased after cisplatin induced AKI in rats	Cisplatin induced AKI in rats	Serum and kidney tissue	Zhu et al., 2016
**MiR-205**	MiR-205 is a marker of ototoxicity in mice treated with aminoglycoside antibiotics	Kanamycin-furosemide stress in mice	Serum	Lee et al., 2018
**Let-7d, MiR-203, and -320**	These 3 miRNAs were significantly changed in urine of rats after drug induced AKI	Gentamicin in rats	Urine	Nassirpour et al., 2014
**11 different miRNAs**	A set of urinary miRNAs was upregulated in a time dependent manner in male Wistar rats	Cisplatin induced AKI in rats	Urine	Pavkovic et al., 2014

I/R, ischemia/reperfusion.

##### MiR-21

MiR-21 is expressed in the kidney of mammals and among others in heart and lung tissue (Lagos-Quintana et al., 2002). It has been analyzed in a large number of preclinical and clinical AKI studies. MiR-21 attenuates I/R injury of the kidney by inhibiting apoptosis (Hu et al., 2014). Suppressing miR-21 expression leads to a reduced activity of tumor necrosis factor α and monocyte chemoattractant protein-1 and thereby renal inflammation (Zhong et al., 2013). A number of studies have shown that miR-21 is differentially expressed in plasma and/or urine in AKI (**Table 1**). However, as miR-21 is expressed ubiquitously, it is likely to be a rather unspecific marker of AKI.

##### MiR-30a and -c

The miR-30 family consists of five highly conserved members termed miR-30a to –e. This family has been shown to be involved in numerous cancers, such as lung cancer, breast cancer, and colorectal cancer (reviewed in (Yang et al., 2017)). In a rat model of AKI, as well as in patients after cardiac surgery, miR-30c-5p was an early diagnostic marker of AKI in urine (Zou et al., 2017). It has, furthermore, been shown that podocyte injury is facilitated by downregulation of the miR-30 family and that this injury can be prevented by the administration of glucocorticoids (Wu et al., 2014).

##### MiR-107

MiR-107 is member of the miR-15/107 family. Members of this family share the AGCAGC near the 5′ end and are involved in several pathways which are crucial in AKI, including stress response and angiogenesis (Finnerty et al., 2010). In patients suffering from septic induced AKI, miR-107 was shown to be elevated in Circulating endothelial cells (Wang et al., 2017).

##### MiR-125b

The miR-125 family is highly conserved between mammals. Of this family, miR-125b has been shown to be involved in ischemia and reperfusion events of several organs, especially the heart (Wang et al., 2014). Additionally, it has been shown that miR-125b is a protective factor in cardiac ischemia and reperfusion injury (Varga et al., 2018). In AKI, miR-125b has been shown to be a potential marker in the diagnosis of ischemic kidney injury (Guclu et al., 2017).

#### Experimental AKI Studies: Therapeutic Potential of miRNAs

In theory, miRNAs bear a fascinating potential not only as diagnostic markers of AKI but also as therapeutics – summarized in the term theranostics. Dysregulated miRNA-expression can be treated *in vivo* and *in vitro* by the application of specific antagonists or agonists vice versa. Obstacles regarding this *in vivo* application of miRNAs are described in the next passage describing the clinical application of miRNAs.

There are a growing number of animal studies showing an effective modulation of miRNA function in the context of AKI. A selection of these studies is summarized in **Table 2**. These data show that AKI severity and recovery from AKI can be influenced by the modulation of miRNAs. However the next step from these observation into humans and finally to clinical practice is by far the biggest one. Obstacles and chances of this step will be further discussed in the next paragraph.

**Table 2:** Selected experimental studies focussing on the therapeutic potential of miRNAs in AKI.

MiRNA	Function	Reference
**MiR-10a-5p, miR-127-3p, -7a, and miR-30a-5p. miR-486-5p**	These miRNAs are carried in EV's from MSC's: pro-regenerative effect in renal recovery from AKI	Tapparo et al., 2019
**MiR-16**	Overexpression of miR-16 in mice attenuates renal function in mice after I/R injury	Chen et al., 2016
**MiR-21**	Via the PTEN/Akt/mTOR pathway miR-21 protects kidneys from I/R injury (LNA-modified anti-miR-21 *in vivo* and *in vitro*)	Song et al., 2018
	Ghrelin leads to an upregulation of miR-21, thereby ameliorating AKI (Ghrelin injection in rats)	Zhang and Shu, 2016
**MiR-107**	MiR-107 antagonist decreased expression of TNF in circulating endothelial cells septic AKI patients and in septic AKI mice.	Wang et al., 2017
**MiR-182-5p**	Inhibition by antisense nucleotides leads to an improved kidney function in a rat model of AKI	Wilflingseder et al., 2017
**MiR-194**	Overexpression of miR-194 mimetic oligonucleotides in HK-2 cells protects from hypoxia and reoxygenation	Shen et al., 2018
**MiR-199a-3p**	Exosomal miR-199a-3b protects against kidney I/R injury *in vitro*	Zhu et al., 2019
**MiR-489**	Mir-489 mimimetic oligonucleotides protect kidneys, whereas antagonism deteriorates renal recovery in mice	Wei et al., 2016
**MiR-668**	MiR-668 is induced *via* HIF-1 in ischemic kidneys and represses mitochondrial protein 18 kDa (MTP18), thereby protects kidneys from ischemic AKI in mice	Wei et al., 2018

EV, extracellular vesicle; MSC, mesenchymal stem cell; I/R, ischemia/reperfusion.

### Clinical Studies Using miRNAs as Biomarkers and Therapeutics

Extensive research activities regarding preclinical as was patient studies have been forwarded regarding the investigation of the role of miRNAs in kidney injury. Taking into consideration the mechanism of action and downstream effects of miRNAs, their therapeutic silencing or overexpression has become a topic of interest in order to target disease activity. Owing to a high degree of intra-species conservation, miRNAs represent perfect target molecules for investigation, since results of animal studies may be easily translated to the human setting to ameliorate or reverse the progression of disease. The identification of the disease and/or tissue/cell-type-specific regulation of miRNAs using transgenic rodent models or pre-clinical therapeutic silencing/overexpression of relevant miRNAs is the prerequisite to understanding pathological events in the kidney and will ultimately results in novel therapeutic strategies to target diseases. Alterations of specific miRNAs in distinct diseases can be perceived to mirror dysregulation of intertwined pathological signalling, because oftentimes miRNAs have equal mRNA target molecules, thus impacting on similar signalling pathways.

MiRNAs have been analyzed in detail as biomarkers of kidney disease (**Table 3**). For instance, several miRNAs (including miR-101-3p, miR-127-3p, miR-210-3p, miR-126-3p, miR-26b-5p, miR-29a-3p, miR-146a-5p, miR-27a-3p, miR-93-3p, and miR-10a-5p) have been shown to be altered in serum samples of patients with AKI (Aguado-Fraile et al., 2015). These novel biomarkers showed a near perfect area under the curve of almost 1. Moreover, miR-210 and -320 were demonstrated to be enriched in blood samples of AKI patients (Lorenzen et al., 2011). Here, miR-210 predicted mortality of AKI patients on the intensive care unit. On the contrary, in patients with terminal kidney failure on renal replacement therapy miR-21 (downregulated) and miR-499 (upregulated) seem to be altered (Neal et al., 2011; Emilian et al., 2012). In patients undergoing cardiac surgery, baseline miR-21 before surgery predicted AKI development after completion of cardiac surgery (Gaede et al., 2016). Another study suggested miR-494 to be highly upregulated in urinary specimens of patients with AKI (Lan et al., 2012). MiR-24 has been demonstrated to drive progression of ischemic AKI (Lorenzen et al., 2014).

**Table 3:** Biomarker studies in humans with kidney disease using miRNAs.

MiRNA	Function	Reference
**miR-101-3p, miR-127-3p, miR-210-3p, miR-126-3p, miR-26b-5p, miR-29a-3p, miR-146a-5p, miR-27a-3p, miR-93-3p, miR-10a-5p**	Biomarker of AKI	Tapparo et al., 2019
**miR-210 and -320**	Biomarker of AKI, predictor of mortality	Chen et al., 2016
**miR-21, miR-499**	Biomarker of End-stage renal disease	Song et al., 2018
**miR-21**	Prediction of AKI development after completion of cardiac surgery	Zhang and Shu, 2016
**miR-494**	Biomarker of AKI in urinary specimens	Wang et al., 2017
**MiR-24**	Biomarker of ischemic AKI	Wilflingseder et al., 2017
**miR-126 and miR-296**	Vesicles derived from endothelial cells containing miR-126 and miR-296 as biomarkers of AKI	Shen et al., 2018
**Mir-30c-5p and -192-5p**	Urinary levels of these two miRNAs are elevated in AKI patients within 2h after admission to an ICU.	Zou et al., 2017
**MiR-107**	MiR-107 is induced in circulating endothelial cells (CEC) of septic AKI patients.	Wang et al., 2017

Contrary to the use of miRNAs as biomarkers, their application as therapeutics is less advanced. A few pre-clinical studies in rodents/cells have investigated the therapeutic potential of miRNAs in AKI (miR-126/miR-296 (Cantaluppi et al., 2012), miR-92a (Henique et al., 2017), miR-709 (Guo et al., 2017)). These preclinical studies suggest that miRNAs may also be applied as powerful therapeutics in the setting of patients with AKI.

However, translation of pre-clinical findings is complicated by several challenges. A major obstacle to transferring animal data to humans is related to the fact that miRNAs are not regulated in a cell-type or organ specific manner. Therapeutic alteration of a specific miRNA may therefore lead to “off-target” changes in unrelated/untargeted organs, which were initially not aimed for. This problem finds it correlate in the fact that merely few investigations involving patients treated with drugs targeting miRNAs have been forwarded to date. The formulation of cell or tissue-specific miRNA drugs by coupling them with tissue-specific antibodies may be a solution to this predicament and could be a focus of future investigations. A major study in patients with kidney disease, which has just entered Phase II is related to a treatment strategy silencing miR-21 in Alport nephropathy patients (clinicaltrials.gov, NCT03373786). In addition to this important study, miR-122 has been silenced effectively in patients with an infection of the hepatitis C virus (Janssen et al., 2013). The use of so-called miRNA-mimics to overexpress miRNAs in patients with malignant pleural mesothelioma and non-small cell lung cancer (clinicaltrials.gov, NCT02369198) has been forwarded. A mimic for miR-34 (MRX34), which acts as a tumor suppressor in a number of cancers was evaluated in a phase 1 clinical trial on liver-based cancers (Beg et al., 2017). This study was terminated early due to the occurrence of adverse events related to immune-mediated mechanisms. An inhibitor against microRNA-17 (miR-17, named RGLS4326), which targets important disease-relevant genes including PKD1 and PKD2, will soon be evaluated as a potential treatment for adult polycystic kidney disease in a phase 1 clinical trial (Lee et al., 2019). (**Table 4**) These clinical studies raise hope that miRNAs may be used as therapeutics in future clinical routine. However, the widespread use of miRNA therapeutics still has a long way to go. Nonetheless, the ongoing and detailed analysis of potential therapeutic use of miRNA modulators in kidney disease represents a fascinating avenue of future investigations and might ultimately improve patient care.

**Table 4:** Clinical trials investigating miRNA modulators.

MiRNA	Clinical setting	Reference
**Anti-miR-21 treatment**	Treatment of Alport nephropathy	Tapparo et al., 2019
**Anti-miR-122 treatment**	Treatment of hepatitis C virus infection	Chen et al., 2016
**Mimic for miR-34 (MRX34)**	tumor suppressor in a variety of cancers was evaluated in a phase 1 clinical trial on liver-based cancers	Song et al., 2018
**Anti-miR-17 treatment (RGLS4326)**	potential treatment for adult polycystic kidney disease	Zhang and Shu, 2016

### Future Outlook Regarding Clinical Studies Using miRNA Modulators in Patients With AKI.

Research into the mechanisms of miRNA-related kidney disease, including AKI, has gained widespread interest over the last decade. The identification and therapeutic applicability of cell- and tissue-specific miRNAs would pave the way for an improvement of the future care of patients. If a disease-specific miRNA is identified, its downstream mechanisms resolved and therapeutic potential proven, it would offer physicians a real alternative for a targeted treatment approach. Aforementioned predicament regarding a lack of tissue-specificity of a number of miRNAs (and still retain its desired function) could be circumvented by a combination of these miRNAs with cell-type specific peptides/antibodies so that the complex may be directed to a tissue of interest.

Several chemistries of miRNA modulators have been described in the past. One of the first studies in the cardiovascular research employed an intravenous treatment approach using Cy3-coupled AntimiRs on a cholesterol based backbone in rodents. This resulted in efficient uptake of the miRNA inhibitor and miRNA suppression (Thum et al., 2008). An additional modification that has been experimentally used includes 2′*O*-methoxyethylphosphorothioate and 2′ fluoro substitutions. These modifications have been shown to lead to specific and strong silencing of the miRNA of interest (Kim, 2005). Furthermore, miRNAs may be silenced by the use of an approach termed as “erasers”. Using this approach a tandem repeat of a sequence that shows perfect complementarity to the miRNA of interest inhibits its function (Sayed et al., 2008).

As an alternative, miRNA silencing can be achieved by use of a locked nucleic-acid (LNA)-based approach. LNAs have been initially tested in a primate model of hepatitis C infection. Here, they have been shown to be highly effective and non-toxic, while also exhibiting a long half-life (Lanford et al., 2010). This resulted in the design and completion of aforementioned clinical study in patients with hepatitis C (Janssen et al., 2013). MiR-122 can be viewed as a special miRNA, since its expression is highly enriched in the liver, thereby making it an ideal candidate for therapeutic interventions, since off-target effects in distant organs are not expected. A locked nucleic acid approach is now widely used and represents the chemistry of choice regarding future clinical use due to its effectiveness, stability, and low profile regarding potential side effects. Almost all miRNA inhibitors are associated with high specificity regarding its target miRNA. A loss of miRNA downregulation may ensue, if a point mutation is detected within the structure of an AntimiR (Krutzfeldt et al., 2007).

A major concern, as stated above, is the fact that the use of miRNA therapeutics may exert off-target effects in nontarget cells and/or organs. In order to circumvent this predicament miRNA modulators may therefore be locally injected in the future. In addition, miRNA modulators may be coupled to cell and/or tissue-specific antibodies/peptides.

In addition to miRNA antagonists a miRNA of interest may also be therapeutically overexpressed by the use of miRNA-mimics (synthetic double stranded precursor miRNA molecules). Efficient recognition and loading of the guide strand into the RNA-induced silencing complex (RISC) is a prerequisite for its use (Martinez et al., 2002).

In conclusion, miRNA modulation in the setting of AKI represents a field of potential major importance and promise in the future. Critically ill patients with AKI on the intensive care unit might greatly benefit, based on pre-clinical studies involving miRNA therapeutics, from miRNA modulation to halt or even reverse the disease process and limit AKI. If proven successful miRNA modulation might save patients from the installation of renal replacement therapy and its associated complications.

## Author Contributions

Both authors wrote the review article.

## Funding

This work was supported by a National Center for Excellence in Research NCCR Kidney.ch Junior grant as well as a grant by the Swiss National Science Foundation (SNSF) to JL.

## Conflict of Interest

TB has received research grants from Bayer AG and Grünenthal GmbH, Germany, and lecture fees from Fresenius Medical Care Germany.

The remaining author declares that the research was conducted in the absence of any commercial or financial relationships that could be construed as a potential conflict of interest.
